# Invasiveness potential of pneumococcal serotypes in children after introduction of PCV13 in Blantyre, Malawi

**DOI:** 10.1186/s12879-023-08022-4

**Published:** 2023-01-26

**Authors:** Amir Kirolos, Todd D. Swarthout, Andrew A. Mataya, Farouck Bonomali, Comfort Brown, Jacquline Msefula, Naor Bar-Zeev, Pui-Ying Iroh Tam, Maaike Alaerts, Sithembile Bilima, Robert S. Heyderman, Neil French

**Affiliations:** 1 Department of Women’s and Children’s Health, Faculty of Health and Life Sciences, Institute of Life Course and Medical Sciences, University of Liverpool, Liverpool, UK; 2 Malawi-Liverpool-Wellcome Research Programme, Blantyre, Malawi; 3 NIHR Mucosal Pathogens Research Unit, Division of Infection and Immunity, Research Department of Infection, University College London, London, UK; 4 Faculty of Medicine, University of Amsterdam, Amsterdam, Netherlands; 5 International Vaccine Access Center, Johns Hopkins Bloomberg School of Public Health, Baltimore, MD USA; 6 Department of Clinical Sciences, Liverpool School of Tropical Medicine, Liverpool, UK; 7 Cardiogenetics Research Group, Center of Medical Genetics, University of Antwerp and Antwerp University Hospital, Antwerp, Belgium; 8 Institute of Infection, Veterinary and Ecological Sciences, University of Liverpool, Liverpool, UK

**Keywords:** Pneumococcus, Invasive Pneumococcal Disease, Acute Respiratory Infection

## Abstract

**Introduction:**

The introduction of PCV13 to the Malawi infant immunization schedule in 2011 has been associated with reduced disease from *Streptococcus pneumoniae*. Improved understanding of serotypes with high invasive potential can guide future vaccination interventions. We aimed to estimate pneumococcal serotypes associated with acute respiratory infection (ARI) and invasive pneumococcal disease (IPD) in hospitalized children in Blantyre, Malawi.

**Methods:**

We analysed data from healthy children under 5 years in the community in Blantyre and children admitted to Queen Elizabeth Central Hospital with ARI between 2015 and 2018. Nasopharyngeal swabs from children were tested for *S. pneumoniae* and serotyped by latex agglutination if positive. We analysed culture-positive blood and cerebrospinal fluid samples from admitted children between 2012 and 2018 to identify cases of IPD after the introduction of PCV13. We calculated the age-adjusted odds ratio (OR) of carriage for *S. pneumoniae* vaccine serotypes (VT) comparing those with ARI to healthy children. We also calculated age-adjusted ORs comparing serotypes causing IPD to carriage in the community with OR > 1 indicating high invasive potential.

**Results:**

Serotypes 5 (OR 24.73 [95% CI 7.90–78.56] p < 0.001), 1 (OR 23.38 [95% CI 9.75–56.06] p < 0.001), and 6B (OR 4.73 [95% CI 1.66–11.64] p = 0.001) had high invasive potential. Serotype 6B was no longer significant (OR 1.34 [95% CI 0.07–6.87] p = 0.777) in a sensitivity analysis accounting for year of recruitment. The prevalence of *S. pneumoniae* carriage in the community was 72.6% [95% CI 71.3–74.0] (3078/4238) and 23.4% (719/3078) of positive community samples were VT. The carriage prevalence in those hospitalised with ARI was 45.5% [95% CI 42.1–48.9] (389/855) and 43.8% of hospital attendees reported antibiotic use prior to admission. We did not identify significant associations with carriage of any serotypes in those with ARI.

**Conclusions:**

Pneumococcal serotypes 5 and 1 are associated with high invasive potential. Despite high community pneumococcal carriage, pre-hospital antibiotic usage likely reduces pneumococcal detection among children admitted in this setting and further research is needed to investigate serotypes associated with ARI. Data from this study can guide future preventative vaccination strategies in Malawi.

**Supplementary Information:**

The online version contains supplementary material available at 10.1186/s12879-023-08022-4.

## Introduction

Pneumonia is the most common cause of death in children aged under 5 years globally and *Streptococcus pneumoniae* (pneumococcus) is the most common cause of bacterial pneumonia in children in sub-Saharan Africa [1–3]. One of the most important preventative public health strategies for *S. pneumoniae* has been the development and introduction of pneumococcal vaccines. In November 2011, Malawi was one of the first African countries to introduce the 13-valent pneumococcal conjugate vaccine (PCV13) to the infant immunisation schedule using a 3+0 regimen (with respective doses at 6, 10 and 14 weeks of age), and a catch-up campaign implemented for children aged up to 1 year. Increased provision of antiretroviral therapy (ART) to people living with HIV has also been associated with a reduced disease burden from *S. pneumoniae* in Malawi [4].

Nasopharyngeal carriage of *S. pneumoniae* is thought to be a pre-requisite for disease and person-to-person spread can occur via carriers in close contact [5]. Despite the high disease burden, *S. pneumoniae* is frequently carried asymptomatically in the nasopharynx of children [6]. A recent study in Karonga, Malawi found evidence of reduced vaccine-type (VT) carriage of *S. pneumoniae* in vaccinated children 3 years after PCV13 introduction but no change in unvaccinated children [7]. It also found evidence of replacement of VT serotypes with non-vaccine serotypes (NVT) in carriers after PCV13 introduction. This is thought to be due to the high background prevalence of pneumococcal carriage contributing to a high force of infection in Malawi [2].

*Streptococcus pneumoniae* can also cause invasive pneumococcal disease (IPD), defined as isolation of *S. pneumoniae* from a sterile site such as blood or cerebrospinal fluid (CSF), associated with severe disease causing bacteraemia and meningitis [8]. Certain *S. pneumoniae* serotypes (1, 3, 5, 7F, 19A) have been previously associated with a higher risk of IPD [9]. Before the introduction of PCV13 in Malawi, serotypes 1, 6A/6B, 14 and 23F were found to be the most common causes of IPD in children between 2004 and 2006 [10]. The introduction of PCV13 in Malawi has been associated with a significant reduction in the incidence of IPD in PCV-vaccinated children but limited evidence of indirect protection benefitting PCV-unvaccinated infants and adults [11]. There has meanwhile been an increase in NVT-IPD since 2015.

Further assessment of both the carriage and invasiveness potential of common serotypes after the introduction of PCV13 can improve understanding of the dominant disease-causing serotypes in Malawi. This is increasingly important with the development of higher-valent vaccines, including the recently approved PCV15 (VAXNEUVANCE™) and PCV20 (Prevnar 20™). We note that while *S. pneumoniae* carriage in acute respiratory infection (ARI) does not assume causation, rates of specific serotype carriage in cases can identify potential associations. We therefore aimed to compare *S. pneumoniae* carriage in healthy children aged under 5 years to those admitted to hospital with ARI. We also aimed to compare serotypes causing IPD with community carriage to identify serotypes associated with higher invasive disease potential.

## Methods

### Study site

Blantyre is the second largest city in Malawi, located in the Southern region, with an urban population of over 900,000 [12]. Queen Elizabeth Central Hospital (QECH) is the government referral hospital providing free medical care to the 1.3 million urban, peri-urban and rural residents and receives children requiring specialist care from throughout Blantyre District. PCV13 coverage is high at over 90% among those age-eligible and is delivered in a 3+0 schedule, with single doses given respectively at 6, 10, and 14 weeks of age. There is a high rate of HIV infection in the population, with an estimated one in five HIV-affected households in Malawi [13]. In 2011, Malawi adopted the WHO recommendation allowing all HIV-infected pregnant or breastfeeding women to commence lifelong antiretroviral therapy regardless of clinical or immunological stage. This has significantly reduced mother-to-child transmission of HIV in recent years.

### Study design

#### Recruitment of cases of ARI

We retrospectively analyzed hospital records and study data collected for children 1–4 completed years of age, admitted to QECH with ARI between 19 June 2015 and 6 December 2018. These participants were enrolled in a previously established long-term prospective surveillance study [11, 14]. We were limited by the original age selection criteria and data on children less than 1 year with ARI were therefore unavailable. Trained nurses based in QECH reviewed paediatric admissions throughout the week to identify eligible participants and seek written informed consent. ARI was defined as a respiratory infection with body temperature measured > 38 °C and a cough of onset within the previous 10 days [15]. Participants had a digital chest x-ray taken and made available to clinicians for routine care. These were later read for study purposes by two independent trained assessors based on WHO criteria for primary endpoint pneumonia. In the case of discordance, the decision of a third assessor was final. For each enrolled child—in addition to routine clinical assessment based on symptoms and imaging, and along with the collection of a nasopharyngeal swab—a brief study-specific case report form with basic demographic information (age, gender, and pre-hospital antibiotic usage) was completed by a parent or guardian.

#### Recruitment of healthy children from the community

Between 19 June 2015 and 6 December 2018, community sampling was conducted during twice annual cross-sectional surveys. These surveys recruited healthy children up to 4 completed years of age (including children under 1 year), based on a cluster randomised sampling frame, to assess pneumococcal carriage as described elsewhere [16]. Households, schools, and vaccination centres were selected from within three non-administrative zones representative of urban Blantyre’s socioeconomic spectrum in medium- to high-density townships. As with the hospital component, written informed consent was sought from the parent/guardian of recruited children.

#### Analysis of IPD

There has been ongoing sentinel surveillance for laboratory confirmed IPD, including bacteraemia and meningitis among all ages, at QECH since 1998 [11, 14]. In accordance with long-standing clinical guidelines, all adults and children presenting to QECH with fever, or clinical evidence of bacteraemia or meningitis undergo blood cultures and, where appropriate, lumbar puncture. Testing is conducted on admitted children as part of routine clinical care. We analysed post-PCV13 data from archived invasive isolates of children aged up to 4 completed years of age admitted to QECH between 2012 and 2018 (including children under one). All children with culture-confirmed *S. pneumoniae* in a blood (bacteraemia) or CSF (meningitis) sample between 1 January 2012 and 31 December 2018 were included in our analysis. We included all post-PCV13 cases from 2012 in this analysis given that laboratory confirmed IPD is a rare outcome, but also conducted a sensitivity analysis looking at cases between 2015 and 2018 to compare directly with the years that healthy children in the community were recruited.

### Sample testing

All hospital ARI and community participants had nasopharyngeal swabs taken. Samples were collected using a Dacron flocked swab, and then immediately placed into 1.5 mL skim milk-tryptone-glucose-glycerol (STGG) medium and processed at Malawi-Liverpool-Wellcome laboratory, co-located to QECH. Samples were tested for *S. pneumoniae* by culture and serotyped by latex agglutination as described elsewhere [16]. This testing methodology allowed the differential identification of PCV13 VT positive samples, but not for differential identification of NVT serotypes; therefore, NVT and non-typeable isolates were classified as NVT serotypes.

Pneumococcal isolates from culture-confirmed blood and CSF samples in children aged under 5 years which grew *S. pneumoniae* on culture between 2012 and 2018 were assessed by the same serotyping methods, as described elsewhere [11, 14].

### Data analysis

Statistical analysis software R (version 4.0.3) was used for data compilation and statistical analyses. We calculated the prevalence of *S. pneumoniae* carriage in the community and in children presenting to hospital with ARI based on the number of nasopharyngeal samples positive for pneumococcus from each setting respectively and calculated exact 95% confidence intervals (CIs). We calculated the proportion (number of a specific serotype/number of total positive pneumococcal samples) and exact 95% CIs of individual serotypes for three groups (healthy children in the community, children admitted with ARI and children with IPD).

We used a logistic regression model to calculate an odds ratio (OR) of carriage, 95% CIs and P value for each serotype by comparing serotype proportions in children with ARI to healthy children in the community. We also used a logistic regression model to calculate an OR, 95% CIs and p value comparing serotypes causing IPD to proportional carriage in the community. We used this OR to determine invasive potential considering OR > 1 as high invasive potential. We included age in our logistic regression models to produce an age-adjusted OR. Given that age was adjusted for in our analysis we included all healthy children in the community (including those aged less than 1 year old) when comparing them with those admitted with ARI.

Using a Bonferroni correction, we considered a p value < 0.002 to be statistically significant based on 26 separate analyses assessing odds ratios for both ARI and IPD cases across 13 VT serotypes.

## Results

### Demographic characteristics

Between June 2015 and December 2018, 4238 healthy children in the community were included in the study. Ages ranged from 28 days to 4 completed years (mean: 2.8 years, median: 3.2 years). Of children recruited in the community, 50.3% were male. Eight hundred and fifty-five children admitted to hospital with ARI were included in the study. Ages ranged from 1 to 4 completed years (mean: 2.1 years, median: 1.8 years) and 57.2% of children with ARI recruited in hospital were male.

Of hospitalised ARI cases, 601 provided information regarding antibiotic use prior to admission and 43.8% (263/601) reported having taken antibiotics prior to attending hospital.

Between January 2012 and December 2018, 149 children aged up to 4 completed years were identified with confirmed IPD (bacteraemia or meningitis). Ages ranged from 2 days to 4 completed years (mean: 1.6 years, median: 1.0 years).

### Pneumococcal carriage prevalence

**Fig. 1:**
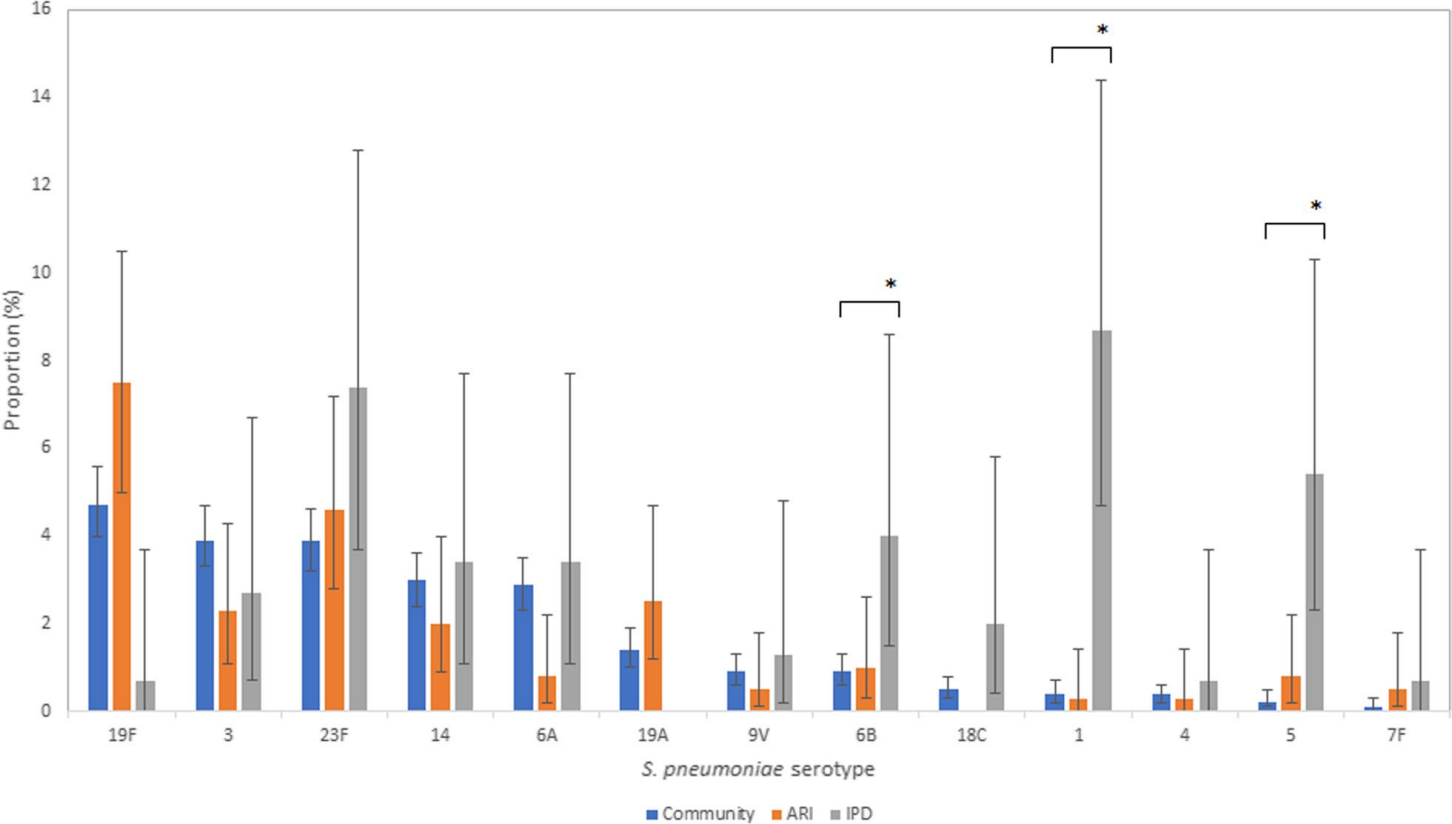
Proportion of *S. pneumoniae* vaccine serotypes in three groups of children (children in the community, with ARI and with IPD). *P value < 0.002 (considered statistically significant based on Bonferroni correction); error bars indicate 95% confidence intervals of the reported proportions

Of 4238 healthy children in the community, the total (VT + NVT) pneumococcal carriage prevalence was 72.6% [95% CI 71.3–74.0] (3078/4238). Of the 3078 samples with pneumococcus 23.4% [95% CI 21.9–24.9] (719/3078) were VT serotypes (Table 1). Serotypes 19F, 3, 23F, 6A and 14 (in descending order of frequency) were the most common VT serotypes carried in healthy children in the community (Fig. 1).

**Table 1:** Number of positive *S. pneumoniae* samples, proportion of total positive samples, odds ratio of carriage by serotype comparing children with ARI to healthy children in the community and odds ratio comparing serotypes causing IPD to carriage in healthy children in the community

Serotype	Community samples		ARI samples		ARI/community samples		IPD samples		IPD/community samples	
	Spn positive (freq)	Proportion (%) [95% CI] (N = 3078)	Spn positive (freq)	Proportion (%) [95% CI] (N = 389)	OR (p value) [95% CI]	Age-adjusted OR (p value) [95% CI]	Spn positive (freq)	Proportion (%) [95% CI] (N = 149)	OR (p value) [95% CI]	Age-adjusted OR (p value) [95% CI]
19F	146	4.7 [4.0–5.6]	29	7.5 [5.0–10.5]	1.62 (0.023) [1.05–2.41]	1.81 (0.007) [1.16–2.75]	1	0.7 [0.0–3.7]	0.14 (0.047) [0.01–0.61]	0.15 (0.061) [0.01–0.69]
3	122	3.9 [3.3–4.7]	9	2.3 [1.1–4.3]	0.57 (0.112)	0.67 (0.266) [0.31–1.28]	4	2.7 [0.7–6.7]	0.67 (0.434) [0.20–1.62]	0.82 (0.699) [0.24–2.01]
23F	120	3.9 [3.2–4.6]	18	4.6 [2.8–7.2]	[0.27–1.08] 1.20 (0.489) [0.70–1.93]	1.29 (0.347) [0.74–2.12]	11	7.4 [3.7–12.8]	1.96 (0.039) [0.98–3.57]	2.06 (0.032) [1.01–3.84]
14	91	3.0 [2.4–3.6]	8	2.0 [0.9–4.0]	0.69 (0.318) [0.31–1.34]	0.66 (0.270) [0.29–1.30]	5	3.4 [1.1–7.7]	1.14 (0.780) [0.40–2.58]	1.06 (0.909) [0.36–2.45]
6A	89	2.9 [2.3–3.5]	3	0.8 [0.2–2.2]	0.26 (0.023) [0.06–0.70]	0.26 (0.023) [0.06–0.70	5	3.4 [1.1–7.7]	1.17 (0.742) [0.41–2.64]	1.19 (0.717) [0.41–2.76]
19A	44	1.4 [1.0–1.9]	10	2.5 [1.2–4.7]	1.82 (0.091) [0.86–3.50]	2.01 (0.061) [0.92–4.01]	0	0	0 (0.979)	0 (0.979)
9V	29	0.9 [0.6–1.3]	2	0.5 [0.1–1.8]	0.54 (0.405) [0.09–1.81]	0.57 (0.444) [0.09–1.95]	2	1.3 [0.2–4.8]	1.43 (0.627) [0.23–4.81]	1.67 (0.498) [0.26–5.91]
6B	27	0.9 [0.6–1.3]	4	1.0 [0.3–2.6]	1.17 (0.766) [0.35–3.02]	1.08 (0.888) [0.31–2.86]	6	4.0 [1.5–8.6]	4.74 (< 0.001)* [1.75–10.93]	4.73 (0.001)* [1.66–11.64]
18C	16	0.5 [0.3–0.8]	0	0	0 (0.986)	0 (0.986)	3	2.0 [0.4–5.8]	3.93 (0.031) [0.91–11.97]	3.67 (0.053) [0.80–12.18]
1	13	0.4 [0.2–0.7]	1	0.3 [0.0–1.4]	0.61 (0.632) [0.03–3.06]	0.81 (0.840) [0.04–4.45]	13	8.7 [4.7–14.4]	22.54 (< 0.001)* [10.16–50.0]	23.38 (< 0.001)* [9.75–56.06]
4	12	0.4 [0.2–0.6]	1	0.3 [0.0–1.4]	0.66 (0.688) [0.04–3.35]	0.81 (0.846) [0.04–4.52]	1	0.7 [0.0–3.7]	1.73 (0.601) [0.09–8.85]	2.76 (0.341) [0.15–15.08]
5	7	0.2 [0.1–0.5]	3	0.8 [0.2–2.2]	3.41 (0.076) [0.73–12.32]	6.09 (0.018) [1.18–25.24]	8	5.4 [2.3–10.3]	24.89 (< 0.001)* [8.82–71.92]	24.73 (< 0.001)* [7.90–78.56]
7F	3	0.1 [0.0–0.3]	2	0.5 [0.1–1.8]	5.30 (0.068) [0.70–32.06]	5.53 (0.082) [0.67–39.03]	1	0.7 [0.0–3.7]	6.93 (0.095) [0.34–54.46]	6.60 (0.132) [0.29–62.57]
Total VT	719	23.4 [21.9–24.9]	90	23.1 [19.0–27.7]	–	–	60	40.3 [32.3–48.6]	–	–
Total NVT	2359	76.6 [75.1–78.1]	299	76.9 [72.3–81.0]	–	–	89	59.7 [51.4–67.7]	–	–
Total	3078		389				149			

Spn: *Streptococcus pneumoniae*; VT: vaccine serotype; NVT: non-vaccine serotype; IPD: invasive pneumococcal disease; ARI: acute respiratory infection; freq: frequency; CI: confidence interval; OR: odds ratio; age-adjusted OR: odds ratio with age included in logistic regression model

*P value < 0.002 considered statistically significant based on Bonferroni correction

Of 855 children admitted to hospital with ARI, the total (VT + NVT) pneumococcal carriage prevalence was 45.5% [95% CI 42.1–48.9] (389/855). Of 389 positive tests, 23.1% [95% CI 19.0–27.7] (90/389) were VT serotypes (Table 1). Serotypes 19F and 23F were the most common VT serotypes carried in children admitted with ARI (Fig. 1).

Among the 855 children admitted with ARI, 111 had confirmed primary endpoint pneumonia on chest x-ray. Of these 111 participants, the total (VT + NVT) pneumococcal carriage prevalence was 39.6% [95% CI 30.5–49.4] (44/111).

### Serotypes associated with ARI and IPD

Table 1 shows the age-adjusted OR of serotype carriage for children admitted with ARI compared to healthy children in the community and serotypes causing IPD compared to community carriage. VT serotypes were a cause of 40.3% [95% CI 32.3–48.6] (60/149) of confirmed IPD cases. Serotypes 5 (OR 24.73 [95% CI 7.90–78.56] p < 0.001), 1 (OR 23.38 [95% CI 9.75–56.06] p < 0.001) and 6B (OR 4.73 [95% CI 1.66–11.64] p = 0.001) were found to have high invasive potential. A sensitivity analysis (Additional file 1: Table S1) looking only at IPD cases between 2015 and 18 found similar results to the main analysis, apart from serotype 6B (OR 1.34 [95% CI 0.07–6.87] p = 0.777) which no longer had a higher invasive potential based on cases restricted to these years.

We did not identify carriage of any VT serotypes significantly associated with ARI compared to their proportional carriage in the community. Children with ARI carrying *S. pneumoniae* had similar proportional NVT (76.9% [95% CI 72.3–81.0]) and VT carriage (23.1% [95% CI 19.0–27.7]) compared to healthy children in the community (76.6% [95% CI 75.1–78.1] and 23.4% [95% CI 21.9–24.9] for NVT and VT respectively). Of the 44/111 positive tests in those with confirmed primary endpoint pneumonia, 31.8% [95% CI 18.6–47.6] (14/44) were VT serotypes. Due to the small number of positive pneumococcal samples in those with primary endpoint pneumonia we were unable to assess carriage of different VT serotypes in this sub-group.

## Discussion

We found serotypes 5 (OR 24.73 [95% CI 7.90–78.56] p < 0.001) and 1 (OR 23.38 [95% CI 9.75–56.06] p < 0.001) had high invasive potential. Our results agree with evidence from previous research [9] and build on evidence from other studies of differing invasiveness potential by pneumococcal serotype [17]. Our results indicate an ongoing risk of invasive disease post-PCV13 from VT serotypes. We didn’t find any serotypes significantly associated with cases of ARI and due to small numbers of children with confirmed primary endpoint pneumonia we were unable to assess serotype carriage in this subgroup. Children with ARI had similar proportional carriage of VT to healthy children in the community. Our analysis is limited given carriage in cases of ARI and pneumonia does not equate to causation. Associations may therefore be harder to detect and further research on whether carriage of specific pneumococcal serotypes are associated with ARI and pneumonia may be of use.

The community carriage prevalence in Blantyre (72.6% [95% CI 71.3–74.0]) is high compared to that of high-income settings [18, 19], but carriage prevalence in those presenting to hospital with ARI was significantly lower (45.5% [95% CI 42.1–48.9]). While antibiotic usage was not asked of healthy children in the community, it is likely to be lower compared to the high reported usage in children presenting to hospital with ARI. Sample collection, handling and processing was the same for both groups and while not definitive, antibiotic usage is the likely cause for this difference. The high background prevalence and rate of pre-hospital antibiotic use suggests that nasopharyngeal swabs have a low predictive value in this setting. While our study did not compare carriage prevalence before and after PCV13 introduction, the prevalence data generated post-PCV13 can be used in future vaccine studies. There is potential to change the current 3+0 schedule of PCV13 in Malawi after studies found lower herd protection effects than expected [2]. One proposal to improve this, based on potential waning effects of the 3+0 vaccine schedule with age, is a 2+1 schedule with two doses in the first three months of life and a booster after 9 months of age [7]. The serotype carriage prevalence data reported in this study can therefore be used to measure the impact of potential schedule changes, as well as following the introduction of new vaccines, on *S. pneumoniae* carriage in Malawi.

A limitation of our study is the absence of PCV13 vaccination status of participants which we were unable to account for in our analysis. We were also limited by the different age distributions in the three groups of children included. We adjusted for age in our models given the younger age profile of those admitted to hospital compared to those sampled in the community. However, the exclusion of children less than 1 year of age with ARI is a limitation given different selection criteria for this hospital recruitment. Given the high rates of pre-hospital antibiotic usage, serotypes with high antibiotic sensitivity may have been underrepresented in positive hospital ARI and IPD samples. However, the similar proportional carriage of NVT and VT serotypes in those with ARI compared to healthy children in the community indicates this may not have affected reported proportional carriage significantly. To improve power, we included all IPD cases post-PCV13 between 2012 and 18 as opposed to the 2015–18 period of community surveillance. While serotype 6B was found to have high invasive potential in our main analysis, our sensitivity analysis of cases from 2015 to 18 found 6B was no longer significantly associated with IPD. The result indicating high invasive potential of 6B should therefore be interpreted with caution given that time and vaccine effects may have altered the main disease-causing serotypes post-PCV13.

Our study found serotypes 5 and 1 had high invasive potential. Data from this study can be used as a baseline for future studies monitoring the ongoing impact of PCV13 and following changes to the vaccine schedule in Malawi.

## Supplementary Information

**Additional file 1: Table S1**. Number of positive *S. pneumoniae* samples, proportion of total positive samples and odds ratio of carriage by serotype comparing children with IPD (2015–2018) to healthy children in the community.

## Data Availability

All data generated or analysed during this study are included in this published article.
