# Supplementary material for: Invasiveness potential of pneumococcal serotypes in children after introduction of PCV13 in Blantyre, Malawi
Source: BMC Infect Dis. 2023 Jan 26;23:56. doi: 10.1186/s12879-023-08022-4 (PMC9881369; doi:10.1186/s12879-023-08022-4)
Supplement: Supplementary file 1 — Additional file 1: Table S1. Number of positive S. pneumoniae samples, proportion of total positive samples and odds ratio of carriage by serotype comparing children with IPD (2015–2018) to healthy children in the community. [file 12879_2023_8022_MOESM1_ESM.docx]

**Additional file 1**

**Table S1** – Number of positive *S. pneumoniae* samples, proportion of total positive samples and odds ratio of carriage by serotype comparing children with IPD (2015-2018) to healthy children in the community

| Serotype | Community samples | |  | IPD (2015-18) samples | |  | IPD (2015-18) / Community samples | |
| --- | --- | --- | --- | --- | --- | --- | --- | --- |
|  | Spn positive (freq) | Proportion (%)  [95% CI]  (N=3078) |  | Spn positive (freq) | Proportion (%)  [95% CI]  (N=389) |  | OR  (p value) | Age-adjusted OR  (p value) |
| 19F | 146 | 4.7  [4.0 - 5.6] |  | 1 | 1.3  [0 - 7.0] |  | 0.26 (0.187)  [0.01 – 1.20] | 0.30 (0.235)  [0.02 – 1.39] |
| 3 | 122 | 3.9  [3.3 - 4.7] |  | 4 | 5.2  [1.4 - 12.8] |  | 1.33 (0.587)  [0.40 – 3.27] | 1.70 (0.322)  [0.50 – 4.29] |
| 23F | 120 | 3.9  [3.2 - 4.6] |  | 5 | 6.5  [2.1 - 14.5] |  | 1.71 (0.255)  [0.59 – 3.92] | 1.87 (0.195)  [0.64 – 4.40] |
| 14 | 91 | 3.0  [2.4 - 3.6] |  | 2 | 2.6  [0.3 - 9.1] |  | 0.88 (0.854)  [0.14 – 2.84] | 0.80 (0.757)  [0.13 – 2.65] |
| 6A | 89 | 2.9  [2.3 - 3.5] |  | 1 | 1.3  [0 - 7.0] |  | 0.44 (0.419)  [0.02 – 2.03] | 0.43 (0.413)  [0.02 – 2.04] |
| 19A | 44 | 1.4  [1.0 - 1.9] |  | 0 | 0 |  | 0 (0.979) | 0 (0.979) |
| 9V | 29 | 0.9  [0.6 - 1.3] |  | 2 | 2.6  [0.3 - 9.1] |  | 2.80 (0.164)  [0.45 – 9.54] | 3.40 (0.111)  [0.53 – 12.50] |
| 6B | 27 | 0.9  [0.6 - 1.3] |  | 1 | 1.3  [0 - 7.0] |  | 1.49 (0.699)  [0.08 – 7.12] | 1.34 (0.777)  [0.07 – 6.87] |
| 18C | 16 | 0.5  [0.3 - 0.8] |  | 2 | 2.6  [0.3 - 9.1] |  | 5.10 (0.032)  [0.80 – 18.36] | 5.02 (0.045)  [0.74 – 20.21] |
| 1 | 13 | 0.4  [0.2 - 0.7] |  | 10 | 13.0  [6.4 - 22.6] |  | 35.19 (<0.001)*  [14.55 – 82.88] | 42.43 (<0.001)*  [15.66 – 113.60] |
| 4 | 12 | 0.4  [0.2 - 0.6] |  | 0 | 0 |  | 0 (0.991) | 0 (0.991) |
| 5 | 7 | 0.2  [0.1 - 0.5] |  | 4 | 5.2  [1.4 - 12.8] |  | 22.04 (<0.001)*  [6.19 – 81.41] | 27.09 (<0.001)*  [6.07 – 107.93] |
| 7F | 3 | 0.1  [0.0 - 0.3] |  | 1 | 1.3  [0 - 7.0] |  | 13.49 (0.025)  [0.66 – 106.70] | 13.29 (0.044)  [0.57 – 135.81] |
| Total VT | 719 | 23.4  [21.9 - 24.9] |  | 33 | 42.9  [31.6 - 54.6] |  | - | - |
| Total NVT | 2359 | 76.6  [75.1 - 78.1] |  | 44 | 57.1  [45.4 - 68.4] |  | - | - |
| Total | 3078 |  |  | 77 |  | | |  |

* p value < 0.002 considered statistically significant based on Bonferroni correction

Spn – *Streptococcus pneumoniae*, VT – vaccine serotype, NVT – non-vaccine serotype, IPD – invasive pneumococcal disease, ARI – acute respiratory infection, freq - frequency, CI –confidence interval, OR – odds ratio, age-adjusted OR – odds ratio with age included in logistic regression model
